# Emerging roles of the long non-coding RNA NEAT1 in gynecologic cancers

**DOI:** 10.1007/s12079-023-00746-x

**Published:** 2023-06-13

**Authors:** Maryam Farzaneh, Mahrokh Abouali Gale Dari, Amir Anbiyaiee, Sajad Najafi, Dian Dayer, Abdolah Mousavi Salehi, Mona Keivan, Mehri Ghafourian, Shahab Uddin, Shirin Azizidoost

**Affiliations:** 1 Fertility, Infertility and Perinatology Research Center, Ahvaz Jundishapur University of Medical Sciences, Ahvaz, Iran; 2 Department of Obstetrics and Gynecology, School of Medicine, Ahvaz Jundishapur University of Medical Sciences, Ahvaz, Iran; 3 Department of Surgery, School of Medicine, Ahvaz Jundishapur University of Medical Sciences, Ahvaz, Iran; 4 Department of Medical Biotechnology, School of Advanced Technologies in Medicine, Shahid Beheshti University of Medical Sciences, Tehran, Iran; 5 Fertility and Infertility Research Center, Kermanshah University of Medical Sciences, Kermanshah, Iran; 6 Cellular and Molecular Research Center, Medical Basic Sciences Research Institute, Ahvaz Jundishapur University of Medical Sciences, Ahvaz, Iran; 7 Department of Immunology, Faculty of Medicine, Ahvaz Jundishapur University of Medical Sciences, Ahvaz, Iran; 8 Translational Research Institute and Dermatology Institute, Academic Health System, Hamad Medical Corporation, 3050, Doha, Qatar; 9 Department of Biosciences, Integral University, Lucknow, Uttar Pradesh 22602 India; 10 Atherosclerosis Research Center, Ahvaz Jundishapur University of Medical Sciences, Ahvaz, Iran

**Keywords:** LncRNAs, NEAT1, Gynecologic cancer, Tumorigenesis, Biomarker

## Abstract

Gynecologic cancers are a worldwide problem among women. Recently, molecular targeted therapy opened up an avenue for cancer diagnosis and treatment. Long non-coding RNAs (lncRNAs) are RNA molecules (> 200 nt) that are not translated into protein, and interact with DNA, RNA, and proteins. LncRNAs were found to play pivotal roles in cancer tumorigenesis and progression. Nuclear paraspeckle assembly transcript 1 (NEAT1) is a lncRNA that mediates cell proliferation, migration, and EMT in gynecologic cancers by targeting several miRNAs/mRNA axes. Therefore, NEAT1 may function as a potent biomarker for the prediction and treatment of breast, ovarian, cervical, and endometrial cancers. In this narrative review, we summarized various NEAT1-related signaling pathways that are critical in gynecologic cancers.

**Graphical abstract:**

**Figure:**
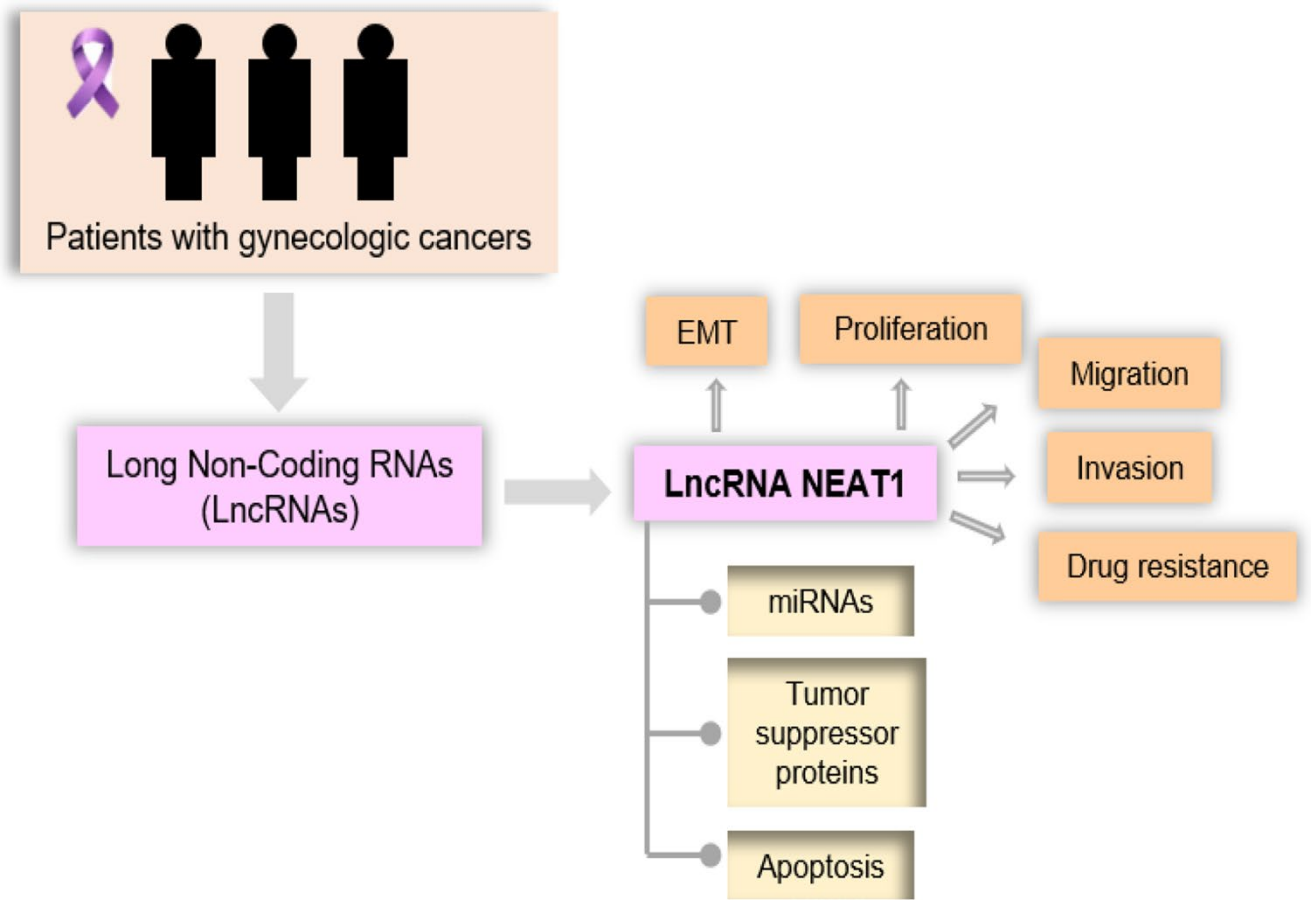
Long non-coding RNA (lncRNA) by targeting various signaling pathways involved in its target genes can regulate the occurrence of gynecologic cancers

## Introduction

Gynecologic cancers, including endometrium, ovaries, cervix, fallopian tubes, and breast are a worldwide problem in women at any age (Kataki et al. 2023). Several factors such as genetics, lifestyle, and exposure to carcinogens may influence the frequency of gynecologic cancers (Razavi et al. 2021). Recently, the molecular biology of cancer has opened a new area of biomedical research (Fiore et al. 2021). The concept of RNA-based cancer therapies has gradually evolved into reality (Zhu et al. 2022). Non-coding RNAs (ncRNAs) have been shown to play important roles in the pathophysiology of different diseases such as cancer by targeting coding information (Balihodzic et al. 2022). ncRNA is classed as either small non-coding RNA (sncRNA) with less than 200 nucleotides (Beg et al. 2022) or long ncRNA (lncRNAs) with more than 200 nucleotides in length. LncRNAs play a significant role in gene transcription, post-transcription, translation, and epigenetic modification (Grammatikakis and Lal 2022). Abnormal expression or malfunction of lncRNAs has been linked to various illnesses (Ni et al. 2022). LncRNAs can influence cell proliferation, apoptosis, migration, and invasion during cancer formation (Yuan et al. 2021). Nuclear enriched autosomal transcript 1 (NEAT1) is a newly discovered lncRNA that plays functional role in cancer carcinogenesis (Zhong et al. 2019). NEAT1 dysregulation has been linked to a poor prognosis in various malignancies (Chen et al. 2017; Thankachan et al. 2021). NEAT1 can bind with multiple downstream miRNAs (sncRNA with approximately 22 nts) and regulate tumor growth and progression (Knutsen et al. 2020). Therefore, targeting NEAT1-miRNAs suggests novel therapeutic targets in gynecologic cancers (Venkatesh et al. 2021; Gu et al. 2022; Hussein et al. 2022). In this review, we summarized several NEAT1/miRNA/mRNA pathways that are critical in the pathogenesis of gynecologic cancers.

## Biological characteristics of NEAT1

- **Structure of NEAT1**

NEAT1 which is known as Nuclear Paraspeckle Assembly Transcript belongs to the lncRNA families and was first defined in 2007 as a thoroughly numerous nuclear RNA (Klec et al. 2019). In humans, the encoded NEAT1 gene is transcribed via RNA polymerase II from the multiple endocrine neoplasia (MEN) type I locus on Chr 11q13.1, and gives rise into two transcriptional isoforms, including a 3756 bp variant as short NEAT1-1 and a 22,743 bp variant as long NEAT1-2 (Zhang et al. 2021; Ronchetti et al. 2020). Both variants share a similar promoter at the 5ʹ-end and a distinct 3ʹ-end which forms two different subtypes (Gu et al. 2022). In opposition to high expression of long NEAT1-2 in the adult mice stomach and intestine, short NEAT1-1 exerts high abundance in a variety of tissues (Prinz et al. 2019a). The main localization site for NEAT1 is the nucleus but it can be detected in the cytoplasm (An et al. 2018).

- **NEAT1 functions in normal tissues**

NEAT1 has been implicated in the formation of nuclear architecture known as paraspeckles for gene transcriptional and splicing programs (Kopp and Mendell 2018). Nuclear speckles constitutes active punctate sections in the nucleus that possess parts of the pre-mRNA spliceosome such as subunits of RNA Pol II, proteins of 3' end processing, small nuclear ribonucleoproteins (snRNPs), SRSFs, m^6^A writers METTL3/14 and reader YTHDC1, and diverse protein kinases that modulate the protein pool in the speckles (Klinge 2018). NEAT1 in correlation with paraspeckle proteins, splicing factor proline/glutamine rich, 54 kDa nuclear RNA- and DNA-binding protein (p54nrb), and paraspeckle component 1 (PSPC1) can provide a structural scaffold (Li et al. 2021a). In contrast to the tendency of NEAT1-1 in microspeckle formation, NEAT1-2 contains a limiting factor to form paraspeckles, thereby nucleus tendency for paraspeckle formation is dependent on NEAT1-2 concentration (Pisani and Baron 2020). The recent knowledge exhibits an overview by which NEAT1 and paraspeckles are associated with cancer. In some instances, NEAT1 could reduce chemoresistance in several biological contents (Pisani and Baron 2020; Shin et al. 2019a). NEAT1 by interaction with gene modulatory signals can induce some variations in gene expression, and stimulate or reduce effective immune responses. Hence, NEAT1 could be a possible predictive or prognostic biomarker (Pisani and Baron 2020). Moreover, the pivotal function of NEAT1 and paraspeckles in the control and modulation of DNA damage repair is well documented, which illustrated a crucial step to preserve cells against genetic damages. Therefore, NEAT1 can indirectly participate in DNA damage repair (Taiana et al. 2020). Also, NEAT1 modulates target genes through assigning and/or arresting transcriptional factors and affecting gene transcription, splicing, RNA stability, and translation (Wang et al. 2020). NEAT1 has been implicated in extracellular matrix remodeling (Wang et al. 2019a; Ruan et al. 2018) and the epigenetic modulatory functions of NEAT1 was reported in different studies (Wang et al. 2019a, b; Dilmaghnai et al. 2021). NEAT1 can regulate histone 3 lysine 9 dimethylation (H3K9me2) at the promoter or interact with DNA methyltransferase 1 (DNMT1) to control cellular behavior (Thankachan et al. 2021; Butler et al. 2019). Given the vital function of paraspeckle in governing gene expression within diverse cellular processes including homeostasis, differentiation, viral infection, immune and stress response along with organ development, NEAT1 may function a necessary role in the specific cellular processes and normal embryonic development (Dong et al. 2018; Bu et al. 2020). During mammary gland development and mammary gland branching morphogenesis, NEAT1-containing paraspeckles is necessary (Standaert et al. 2014).

**Fig. 1:**
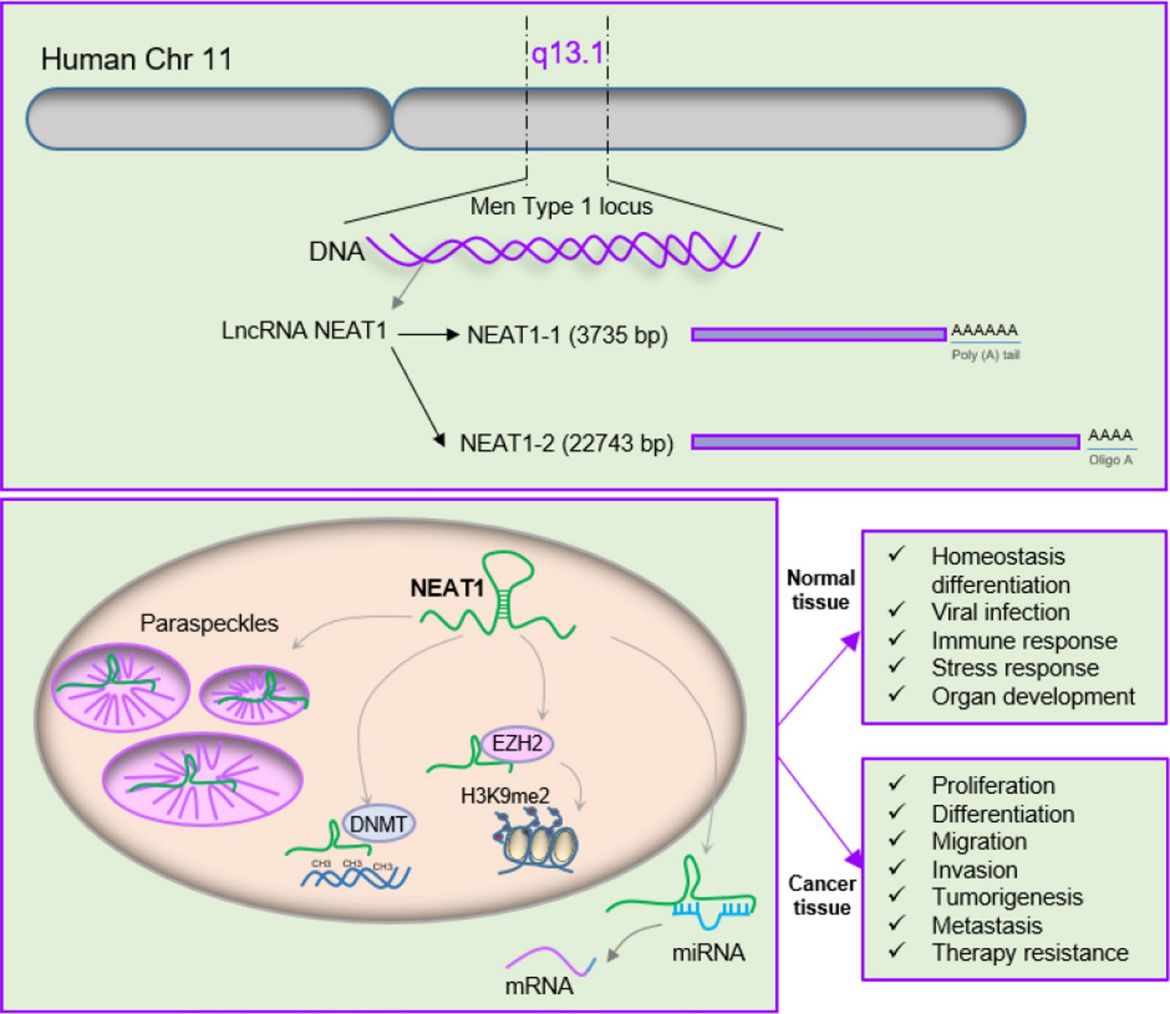
Biogenesis and function of lncRNA NEAT1: In humans, the encoded NEAT1 gene is transcribed via RNA polymerase II from the multiple endocrine neoplasia (MEN) type I locus on Chr 11q13.1 and gives rise into two transcriptional isoforms, including a 3756 bp variant as short NEAT1-1 and a 22,743 bp variant as long NEAT1-2. NEAT1 can function as a scaffold for multiple RNA-binding proteins (RBP), paraspeckle, guide, and competing endogenous RNA (ceRNA). NEAT1 as a ceRNA prevents miRNAs interactions with their downstream targets. Besides, NEAT1 as a guide can bind with RNA-binding proteins to help their correct localization at a specific gene locus

NEAT1 as a guide can bind with RNA-binding proteins and chromatin-modifying complexes, and help them localize at their transcriptional loci (Statello et al. 2021; Fang and Fullwood 2016). Recent studies have identified that NEAT1 as a competing endogenous RNA (ceRNA) can sponge or suppress miRNAs and prevent their effect on mRNAs (Meng et al. 2021; López-Urrutia et al. 2019). Therefore, NEAT1 can function as a scaffold, paraspeckle, guide, and ceRNA. Figure 1 shows the structure and functions of NEAT1.

- **NEAT1 functions in cancer cells**

NEAT1 shows typical features of tumor drivers because it can be involved in cancer initiation and progression, and its abnormal expression is associated with clinical characteristics including metastasis, therapy resistance, and patient survival (Snyder et al. 2022; Knutsen et al. 2022). Evidence accumulation revealed that NEAT1 can promote stem cell properties and mediate the oncogenic phenotype in different malignancies (Li et al. 2021a; Moreno-García et al. 2020). In reproductive system tumors, this lncRNA appears to regulate EMT processes and tumor metastasis by targeting various miRNAs, RNA binding proteins, mature mRNAs, and non-protein coding genes (Li et al. 2021b; Luo et al. 2020).

- **NEAT1 in non-cancerous disorders**

There is emerging proof that NEAT1 can function as a miRNA sponge and interact with target mRNAs during the progression of non-cancerous disorders (Prinz et al. 2019a, b). It has been confirmed that direct binding of NEAT1 to miR-27b-3p repressed diabetic nephropathy (Wang et al. 2019a). NEAT1 by interaction with miR-339-5p has been implicated in mesangial gene expression and functions in diverse diabetic-correlated injury models (Reichelt-Wurm et al. 2022). A recent study on acute kidney injury (AKI) observed that high expression of Neat1-2 by suppressing miR-129-5p induced kidney injury and apoptosis (Ma et al. 2022). In a study on patients with acute ischemic stroke, high expression of NEAT1 was found to inhibit miRNA‐124 and miRNA‐125, and triggered the expression of TNF‐α, IL‐6, IL‐8, and IL‐22 (Li et al. 2020a). Another study observed that NEAT1 had the capacity to target miRNAs in atherosclerotic cardiovascular diseases. Direct binding of NEAT1 to miR-128 promoted inflammation and oxidative stress in atherosclerosis progression (Chen et al. 2019). In diabetic animal models, high expression of NEAT1 deteriorated myocardial ischemia reperfusion injury through induction of apoptosis and autophagy (Ma et al. 2018). Recent finding confirmed the role of NEAT1 in modulating the immune responses of retinal Müller cell to Toxoplasma gondii (Rochet et al. 2019). In a viral infection, gel containing NEAT1-siRNA reduced the activity of inflammatory macrophage and promoted HTNV propagation (Yang et al. 2022). Therefore, NEAT1 has a pivotal function in chronic diseases and future researches will assist to figure out the complete story of such intriguing lncRNA.

## Functional roles of NEAT1 in gynecologic cancers

**Fig. 2:**
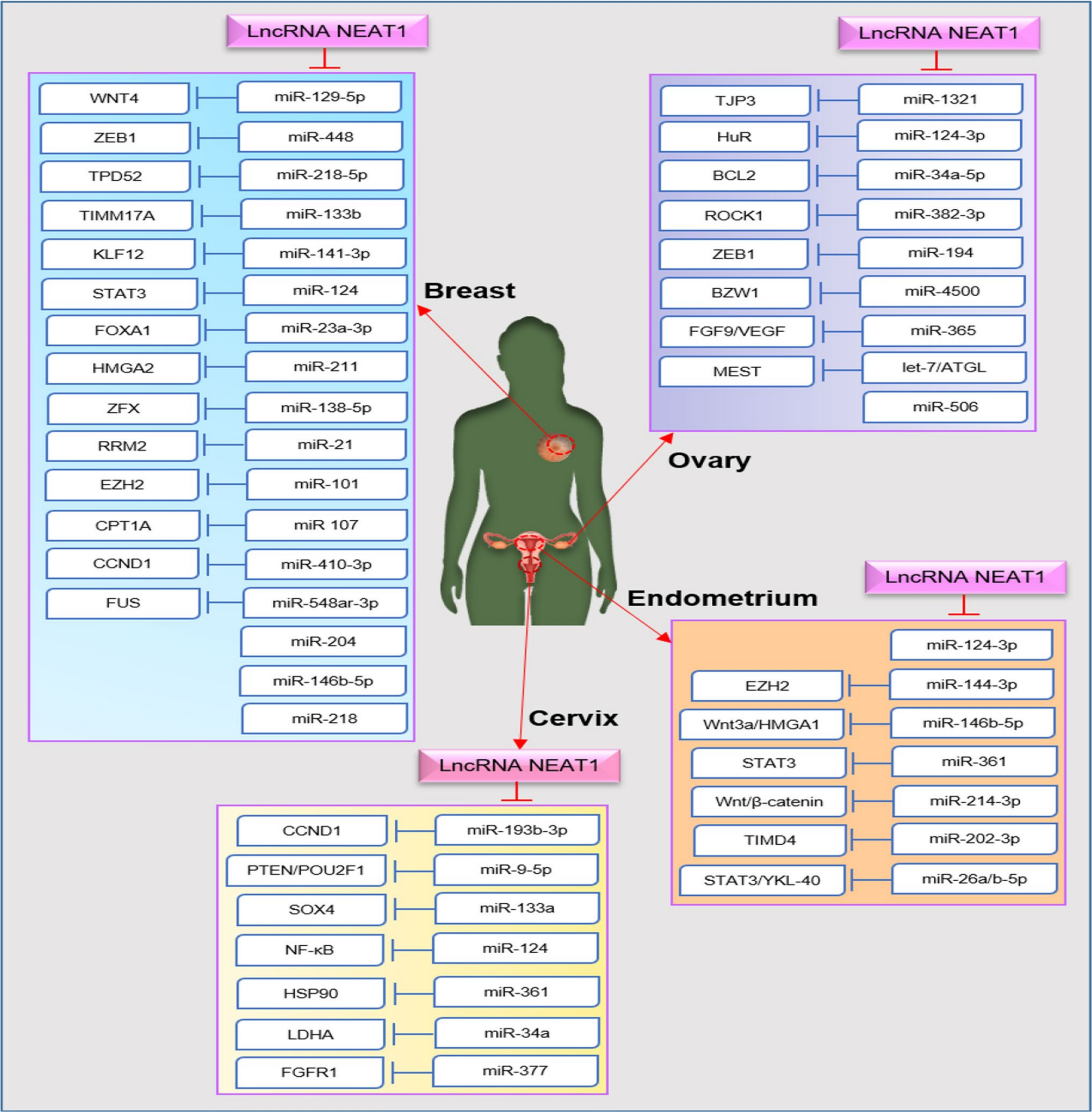
LncRNA NEAT1 functions in gynecologic cancers. NEAT1 has the potential to interact with several miRNAs/mRNAs axes and regulate the tumorigenesis of gynecologic cancers such as breast, cervical, ovarian, and endometrium

It has been reported that NEAT1 by targeting various axes plays a role in the pathogenesis of gynecologic cancers (Table 1). Here, we listed several NEAT1/miRNA/mRNA axes that are essential in breast, cervical, ovarian, and endometrial cancers (Fig. 2).

**Table 1:** The role of lncRNA NEAT1 in gynecologic cancers

Cancer	Target		Results		Refs.
	Suppression	Stimulation	Increase	Decrease	
Breast	miR-204	–	Tumor cell proliferation	Apoptosis	Müller et al. (2019)
	miR-129-5p	WNT4	Tumor cell growth, colony formation, stemness, and malignancy	–	Lo et al. (2016)
	miR-448	ZEB1	Tumor cell proliferation, migration, and invasion	–	Jiang et al. (2018)
	miR-218-5p	TPD52	Tumor cell proliferation, and migration	–	Ren et al. (2021)
	miR-218	–	Tumor cell proliferation, and invasion	–	Zhao et al. (2017)
	miR-133b	TIMM17A	Tumor cell migration, and invasion in vitro and tumor metastasis in vivo	–	Li et al. (2019)
	miR-141-3p	KLF12	Tumor cell proliferation, migration, invasion, and in vivo metastasis	Chemosensitivity to cisplatin, paclitaxel and 5-fluorouracil	Zhou et al. (2021)
	miR‑124	STAT3	Tumor cell proliferation	Cell cycle arrest	Pang et al. (2019)
	miR-146b-5p	–	Tumor cell proliferation, migration, and invasion	–	Li et al. (2020b)
	miR-410-3p	CCND1	Tumor cell proliferation, migration, invasion, and EMT	–	Liu et al. (2020)
	miR-548ar-3p	FUS	Tumor cell proliferation	Apoptosis	Ke et al. (2016)
	miR-23a-3p	FOXA1	Tumor cell proliferation	Chemosensitivity to Taxol	Zhu et al. (2021)
	miR-211	HMGA2	Tumor cell proliferation, migration, invasion, and EMT phenotype	Apoptosis Chemosensitivity to 5-FU	Li et al. (2017)
	miR-138-5p	ZFX	Tumor cell proliferation, migration, and invasion in vitro and tumor growth in vivo	Apoptosis	Yao et al. (2020)
	miR-21	RRM2	Tumor cell proliferation and migration	–	Quan et al. (2020)
	miR-101	EZH2	Tumor cell proliferation and DNA synthesis	–	Qian et al. (2017)
	miR-107	CPT1A	Tumor cell proliferation, migration, and invasion	Apoptosis	Xiong et al. (2019)
Cervix	miR-193b-3p	CCND1	Tumor cell proliferation, colony formation, and radio-resistant	Apoptosis and cell cycle arrest in G0/G1 phase	Han et al. (2018)
	–	Cyclin D1, CDK4, AKT/PI3K, MMP2	Tumor cell proliferation and invasion	Apoptosis, caspase 3	Guo et al. (2018)
	miR-9-5p	PTEN and POU2F1	Tumor cells initiation, proliferation, and migration	–	Xie et al. (2019)
	miR-133a	SOX4	Tumor cell proliferation, colony formation, migration, and invasion	–	Wang et al. (2019c)
	miR-124	NF-κB, N-cadherin, MMP-2, MMP-9, Vimentin	Tumor cell proliferation, migration, invasion, EMT, TNM stage, and lymph node metastasis	Apoptosis	Shen et al. (2020)
	miR-361	HSP90	Tumor cell invasion, sphere formation, and EMT	–	Xu et al. (2020a)
	miR-34a	LDHA	Tumor cell growth and proliferation	The sensitivity of 5-Fu resistant	Shao et al. (2021)
	miR-377	FGFR1	Cell viability, survival, and migration	Apoptosis	Geng et al. (2022)
Ovary	miR-1321	TJP3	EMT, invasion and migration	–	Luo et al. (2020)
	miR-124-3p	HuR	Tumor cell proliferation and invasion, the FIGO stage, and lymph node metastasis	–	Chai et al. (2016)
	miR-34a-5p	BCL2	Tumor cell proliferation	Apoptosis and caspase-3	Ding et al. (2017)
	miR-382-3p	ROCK1	Metastasis, migration, and invasion	–	Gan et al. (2018)
	miR-506	–	Tumor cell growth, proliferation, progression, and cell–cell adhesion	–	Yong et al. (2018)
	miR-194	ZEB1	EMT and drug-resistant phenotype	The sensitivity to PTX	An et al. (2017)
	miR‑4500	BZW1	Cell proliferation, colony formation, migration, and glycolysis	Apoptosis	Xu et al. (2020b)
	miR-365	FGF9, VEGF	Cell proliferation, colony formation, and angiogenesis	–	Yuan et al. (2021)
	let-7, ATGL	MEST	Cell proliferation, migration, and invasion	–	Yin and Wang (2021)
Endometrium	miR-124-3p	–	Tumor cell proliferation, migration, and invasion	Apoptosis	Yuan et al. (2022)
	miR-146b-5p	Wnt3a/HMGA1	Tumor cell proliferation, migration, and invasion	–	Huang et al. (2019)
	miR-144-3p	EZH2	Tumor cell proliferation, migration, and invasion	–	Wang et al. (2019c)
	miR-361	MEF2D, ROCK1, WNT7A, STAT3, VEGFA, PDE4B and KPNA4	Tumor cell proliferation, invasion, and sphere formation	TX responsiveness	Dong et al. (2019)
	miR-214-3p	Wnt/β-catenin, HMGA1, c-myc, MMP9	Tumor cell proliferation, motility, and invasion	–	Wang et al. (2017)
	miR-202-3p	TIMD4	Tumor cell proliferation, migration, and invasion	Apoptosis	Xu et al. (2020c)
	miR-26a/b-5p	STAT3/YKL-40	Tumor cell growth, metastasis, and progression	–	Fan et al. (2021)

### Breast cancer

- **NEAT1/miR-204**

A study suggested that serum levels of NEAT1 along with lncRNA H19 were upregulated in plasma samples of breast cancer patients compared with healthy women. In lymph node‐positive and TNBC patients, high expression of NEAT1 was observed. The inhibition of NEAT1.1 and NEAT1.2 strongly suppressed cell proliferation and enhanced apoptosis in MCF-7 breast cancer cells. miR‐204 as a tumor suppressor was found to reduce the stimulatory effect of NEAT1 on cell proliferation. Therefore, siRNA NEAT1.1 and miR-204 mimic enhanced cell apoptosis. Also, the apoptosis-inductive effect of miR-204 overexpression increased in the presence of Camptothecin (CPT) treatment (Müller et al. 2019).

- **NEAT1/miR-129-5p/WNT4**

Upregulation of NEAT1 was reported to stimulate breast tumorigenicity by targeting the breast cancer susceptibility gene 1 (BRCA1) protein. BRCA1 is a tumor suppressor protein that regulate cell differentiation and tumorigenesis. Besides, NEAT1 could block miR-129-5p expression and upregulate WNT4 levels as a target of miR-129-5p. WNT4 is an oncogenic factor through the WNT signaling. Therefore, NEAT1 was necessary for the malignancies of breast cancer cells (Lo et al. 2016).

- **NEAT1/miR-448/ZEB1**

Jiang et al. (2018) showed that NEAT1 by sponging miR-448 downregulated its levels, and consequently increased the expression of zinc finger E-box binding homeobox 1 (ZEB1). ZEB1 is a pivotal transcription factor during EMT process and breast cancer progression (Wu et al. 2020). NEAT1 downregulation was shown to suppress cell proliferation in MCF-7, MDA-MB-453, MDA-MB-231, and SKBR3 breast cancer cells. Also, miR-448 mimic showed the same effect on the proliferative potentials of cancer cells. Consistent with the silencing results, NEAT1 overexpression promoted cancer cell proliferation, migration, and invasion through repressing miR-448 and enhancing ZEB1 expression.

- **NEAT1/miR-218-5p/TPD52**

Ren et al. (2021) demonstrated that NEAT1 suppressed miR-218-5p and accelerated the expression of tumor protein D52 (TPD52), which is already known to be upregulated in breast cancer (Wang et al. 2022), and is correlated with worse clinicopathological features in breast cancer patients (Roslan et al. 2014). The NEAT1/miR-218-5p/TPD52 axis was shown to promote cell proliferation and migration of breast cancer cells. TPD52 overexpression has the same effects on the proliferative and migratory capacities of cancer cells, while its downregulation inhibited tumor growth in vivo.

- **NEAT1/miR-218**

Zhao et al. (2017) evaluated the stimulatory effects of NEAT1 on breast cancer cell proliferation and invasion via repressing miR-218. NEAT1 knockdown reduced the proliferation of MCF-7 or SK-BR-3 cells in colony formation assay, while simultaneous transfection of cancer cells with small interfering RNA against NEAT1 (si-NEAT1) and miR-218 inhibitor showed an opposite effect on cell proliferation. The transwell cell invasion assay showed that NEAT1 knockdown inhibited the invasive capacity of cancer cells, while co-transfection revealed reverse impact.

- **NEAT1/miR-133b/TIMM17A**

Li et al. (2019) demonstrated that miR-133b expression was significantly decreased in more than 92% of breast cancer tissues. This miRNA was also higher in tissues of breast cancer patients compared to the normal adjacent tissues, and induced worse clinicopathological features such as tumor histological and lymph node metastasis in breast cancer patients. miR-133b can interact with NEAT1 and overexpression of miR-133b and NEAT1 silencing inhibited migration and invasion of MCF-7 and MDA-MB-231 cells. In contrast, miR-133b silencing or NEAT1 overexpression repressed the inhibitory effects on migratory and invasive capacity of cancer cells. Additionally, miR-133b silencing was shown to enhance the oncogenic features of cancer cells and tumor metastasis in vivo via targeting the mitochondrial protein translocase of inner mitochondrial membrane 17 homolog A (TIMM17A), which is already known to promote aggressiveness in the breast cancer cells (Yang et al. 2016).

- **NEAT1/miR-141-3p/KLF12**

Zhou et al. (2021) demonstrated high levels of NEAT1 in extracellular vesicles (EVs) extracted from serum samples of breast cancer patients compared to healthy subjects. NEAT1 was shown to enhance the proliferation, migration, and invasion, and promote the chemoresistance of MCF-7 and MDA-MB-231 breast cancer cells to cisplatin, paclitaxel, and 5-fluorouracil in vitro, while NEAT1 silencing reversed these effects. NEAT1 was found to sponge miR-141-3p and consequently upregulated the expression of Krüppel-like factor 12 (KLF12) to regulate breast cancer progression.

- **NEAT1/miR‑124/STAT3**

NEAT1 was shown by Pang et al. to be upregulated in breast cancer cells and tissues, and inhibited miR-124 expression (Pang et al. 2019). NEAT1 overexpression enhanced the proliferation of MCF-7 cells and accelerated cell cycle progression. NEAT1 knockdown inhibited proliferation and caused G0/G1 cell cycle arrest in cancer cells. miR-124 was shown to target the signal transducer and activator of transcription 3 (STAT3). To assess the effect of miR-124 on the malignant features of cancer cells, MTT assay and cell cycle analysis revealed that miR-124 overexpression inhibited cell proliferation and the G0/G1 phase cell cycle arrest, while STAT3 overexpression reversed this effect in breast cancer cells.

- **NEAT1/miR-146b-5p**

Li et al. (2020b) found that NEAT1 is upregulated in BT474, MCF-7, MDA-MB-231, MDA-MB-453, and SK-BR-3 breast cancer cells relative to MCF10A human breast cell line and in 56 breast cancer tissues compared to adjacent non-tumor tissues. High expression level of NEAT1 was significantly associated with decreased overall survival. NEAT1 silencing was found to reduce cell proliferation and colony formation, decrease EMT, migration, and invasion of MDA-MB-453 cells. By contrast, NEAT1 overexpression enhanced these oncogenic features. Furthermore, miR-146b-5p was shown to be negatively regulated by NEAT1 and its overexpression reversed the oncogenic impacts of NEAT1 on cell proliferation and EMT.

- **NEAT1/miR-410-3p/CCND1**

Liu et al. (2020) revealed that miR-410-3p (among 5 screened miRNAs) expression downregulated in BT549, MDA-MB-231, ZR- 229 75-30, SKBR3, and MCF7 cells compared to MCF-10A cells, and also in breast cancer tissues compared to normal adjacent tissues. NEAT1 was observed to block miR-410-3p and stimulate cell proliferation, migration, invasion, and EMT. Among 5 screened target genes for miR-410-3p (HMGB1, STAT3, IRS1, SOX2, and CCND1), the oncogene encoding cyclin D1 (CCND1) had the most expression level in breast cancer tissues. CCND1-encoded cyclin D1 was known to be upregulated in breast cancer tissues (Adorno-Cruz et al. 2021). Therefore, NEAT1 by targeting the miR-410-3p/CCND1 axis could improve the progression of breast cancer.

- **NEAT1/miR-548ar-3p/FUS**

Ke et al. (2016) reported that NEAT1 knockdown suppressed proliferation of MDA-MB231 cells, and induced apoptosis and increased the percentage of apoptotic cells. RNA immunoprecipitation revealed that NEAT1 interacted with the RNA binding protein (RBP) Fused in Sarcoma (FUS) and repressed miR-548ar-3p. Therefore, miR-548ar-3p overexpression downregulated NEAT1 and triggered apoptosis in breast cancer cells.

- **NEAT1/miR-23a-3p/FOXA1**

Zhu et al. (2021) demonstrated that NEAT1 was upregulated in breast cancer cell lines and tissues compared to MCF10A benign breast cell line and non-tumorous tissues. NEAT1 silencing suppressed cell proliferation in MDA-MD-231 and SKBR3 cells, and enhanced chemosensitivity to Taxol. RNA-pull down and luciferase assays demonstrated that NEAT1 could bind with miR-23a-3p and trigger the expression of Forkhead Box A1 (FOXA1). miR-23a-3p expression was observed to be downregulated in 40 breast cancer tissues compared with adjacent non-cancerous tissues.

- **NEAT1/miR-211/HMGA2**

Li et al. (2017) illustrated that NEAT1 upregulation was associated with poor prognosis in breast cancer patients. NEAT1 knockdown suppressed proliferation, migration, and invasion, and reversed the EMT phenotype of MDA-MB-231 and MCF-7 cells, and induced apoptosis. Additionally, NEAT1 downregulation improved chemosensitivity to 5-FU in breast cancer cells. Among several predicated miRNAs with potential interaction with NEAT1, miR-211 was confirmed in the luciferase reporter assay. Moreover, the high mobility group A2 (HMGA2) gene was found as a downstream gene of NEAT1. miR-211 mimic inhibited cell migration, invasion, and EMT phenotype of MDA-MB-231 and MCF-7 cells. Besides, HMGA2 knockdown showed the same effect with NEAT1 silencing. NEAT1 knockdown decreased metastasis through the smaller number of lung metastatic colonies compared with the control animals.

- **NEAT1/ miR-138-5p/ZFX**

Yao et al. (2020) reported that NEAT1 expression was increased in breast cancer tissues and SUM-185, MCF-7, and T47D breast cancer cells. Bioinformatics evaluations using the starBase v3.0 tool predicted miR-138-5p as a target of NEAT1. NEAT1 by blocking miR-138-5p enhanced the proliferation, migration, and invasion of cancer cells, and inhibited apoptosis. Zinc finger protein X-linked (ZFX) was identified as a target gene of miR-138-5p, which can be overexpressed by NEAT1 in breast cancer cells. Overexpression of miR-138-5p demonstrated the same effects as NEAT1 knockdown..

- **NEAT1/miR-21/RRM2**

Quan et al. (2020) suggested that the NEAT1/miR‐21/RRM2 axis can be involved in breast cancer progression. The Kaplan–Meier curve revealed that breast cancer patients with high expression levels of NEAT1, miR‐21, and ribonucleotide reductase regulatory subunit M2 (RRM2) had a worse prognosis compared with those with low levels. Importantly, overexpression of miR‐21 was found to promote tumor cell proliferation and migration.

- **NEAT1/miR-101/EZH2**

Qian et al. (2017) demonstrated upregulation of NEAT1 in 43 paired breast cancer samples cell lines. They showed that NEAT1 knockdown reduced the proliferation and DNA synthesis of MCF-7 and MDA-MB-453 cells compared to the control group. Also, among several miRNAs with abnormal expression in the past studies (miR-34a, miR-101, miR-761, miR-320b, miR-9, miR-214, and miR-107), miR-101 showed the most significant change by 7.5 times compared to the control group. Consistent with this result in breast cancer cells, patients’ tissues also demonstrated significant downregulation of miR-101 compared to adjacent non-tumor tissues. miR-101 is a target of NEAT1 in breast cancer cells and Enhancer of Zeste 2 Polycomb Repressive Complex 2 Subunit (EZH2) was identified as a downstream target of miR-101. The expression of EZH2 was positively correlated with NEAT1 levels. NEAT1 knockdown reduced proliferation of MCF-7 and MDA-MB-453 cells, unlike miR-101 knockdown promoted cancer cell growth. Also, the BrdU assay revealed reduced DNA synthesis upon NEAT1 knockdown, unlike miR-101 inhibition.

- **NEAT1/miR-107/CPT1A**

Xiong et al. (2019) primarily analyzed the expression levels of NEAT1 and miR-107 in MCF-7 and MDA-MB-231 breast cancer cells compared with MCF-10A cells. The results showed increased expression of NEAT1 versus downregulated miR-107 in breast cancer cells. NEAT1 knockdown increased miR-107 expression, and suppressed cell proliferation, migration, and invasion in breast cancer cells. Carnitine Palmitoyltransferase 1A (CPT1A) was identified as the downstream target gene of miR-107. miR-107 was shown to negatively regulate NEAT1 expression and regulate breast cancer progression through affecting the tumor development-associated genes, including TIMP-1, PDGF-A, SERPINB2, cyclin D1, CDK4, and CPA1A in breast cancer cells. In breast cancer cells, NEAT1 can interact with miR-107 and regulate the expression of CPT1A.

### Cervical cancer

- **NEAT1/miR-193b-3p/CCND1**

High expression of NEAT1 was identified in non-sensitive and radio-resistant cervical cell lines. NEAT1 was found to enhance cell proliferation and colony formation. While the knockdown of NEAT1 triggered apoptosis and cell cycle arrest in the G0/G1 phase. NEAT1 by sponging miR-193b-3p could regulate CCND1 expression. Therefore, NEAT1 induced cervical cancer cell radio-resistance of via competitively binding miR-193b-3p to up-regulate the expression of CCND1 (Han et al. 2018).

- **NEAT1/AKT/PI3K**

It has been shown that high expression of NEAT1 increased the proliferation and invasion of cervical cancer cells by targeting cyclin D1, CDK4, AKT/PI3K, and matrix metallopeptidase 2 (MMP2). NEAT1-siRNA transfection was reported to suppress cell proliferation and trigger apoptosis by targeting caspase 3. Therefore, NEAT1 through regulating the AKT/PI3K signaling may play important role in cervical cancer diagnosis and treatment (Guo et al. 2018).

- **NEAT1/miR-9-5p/PTEN/POU2F1**

A recent study revealed that NEAT1 participated in initiation and progression of both cervical cancer tissues and cell lines by binding to miR-9-5p and suppressing its expression. High expression of NEAT1 stimulated cervical cancer cells proliferation and migration by targeting the phosphatase and tensin homolog (PTEN) and POU class 2 homeobox 1 (POU2F1) protein coding genes (Xie et al. 2019).

- **NEAT1/miR-133a/SOX4**

NEAT1 was explored to stimulate cervical cancer development by blocking miR-133a expression. that NEAT1 via targeting SRY-Box transcription factor 4 (SOX4) as a downstream target of miR-133a can induce colony formation, cell proliferation, migration, and invasion in cervical cancer cells. SOX4 plays essential roles in cancer progression and metastasis (Pan et al. 2022). Knockdown of NEAT1 by siRNA reduced cervical cancer cell migration and invasion capacity. Therefore, the NEAT1/miR-133a/SOX4 axis presented an important role in the pathogenesis of cervical cancer (Wang et al. 2019c).

- **NEAT1/miR-124/NF-κB**

An experiment reported that NEAT1 expression was increased in cervical cancer tissues and HeLa and SiHa cell lines. NEAT1 could accelerate tumor cell proliferation, migration, invasion, EMT, TNM stage, and lymph node metastasis. Overexpression of NEAT1 stimulated the nuclear factor kappa B (NF-κB) pathway by blocking miR-124. NF-κB is a transcriptional factor that plays a crucial role in cell proliferation and migration. miR-124 was found to reduce tumor cell migration, invasion, and EMT by suppressing N-cadherin, MMP-2, MMP-9, Vimentin, and p-NF-κB p65 (Shen et al. 2020).

- **NEAT1/miR-361/HSP90**

A recent study detected that NEAT1 functions as an oncogene in patients with cervical cancer and induced worse survival. NEAT1 could stimulate invasion, sphere formation, and EMT in cervical cells by sponging miR-361. miR-361 by targeting E-cadherin and Zonula occludens-1 (ZO-1) was positively associated with patient survival. NEAT1 was shown to increase heat shock protein 90 (HSP90) expression as a downstream target of miR-361 and a key EMT activator (Xu et al. 2020a).

- **NEAT1/miR-34a/LDHA**

NEAT1 was shown to be upregulated considerably in cervical cancer tissues and cell lines. NEAT1 was found to be linked to 5-Fu resistance. Furthermore, in 5-Fu resistant CaSki cervical cancer cells, NEAT1 expression was considerably increased. NEAT1 knockdown by shRNA significantly improved the sensitivity of 5-Fu resistant CaSki cells. Furthermore, in cervical cancer patient tissues, there was a negative connection between NEAT1 and miR-34a. Therefore, NEAT1 suppression increased the sensitivity of cervical cancer cells to 5-Fu through the miR-34a/LDHA pathway (Shao et al. 2021).

- **NEAT1/miR-377/FGFR1**

In cervical cancer cells, NEAT1 and FGFR1 expression levels were significantly higher, whereas miR-377 expression was significantly reduced. After NEAT1 knockdown, miR-377 expression was elevated, cell viability and migration were decreased, and apoptosis was triggered in HeLa and C33A cells. Low expression of FGFR1 decreased cell survival and migration, and caused apoptosis. Through regulation of the miR377/FGFR1 axis, inhibition of NEAT1 decreased cell survival and migration, and accelerated apoptosis in cervical cancer cells (Geng et al. 2022).

### Ovarian cancer

- **NEAT1/miR-1321/TJP3**

In ovarian cancer, high expression of NEAT1 as an independent factor was positively correlated with tumor grade, distant metastasis, and poor prognosis (Chen et al. 2016). NEAT1 was reported to mediate the expression of tight junction protein three (TJP3) and repress the function of miR-1321, thereby promoting EMT, invasion, and migration of ovarian cancer cells (Luo et al. 2020).

- **NEAT1/miR-124-3p/HuR**

In ovarian cancer patients and cell lines, overexpression of NEAT1 induced the FIGO stage and lymph node metastasis. It has been displayed that NEAT1 by sponging miR-124-3p and binding to the human antigen R (HuR) protein could stimulate tumor cell proliferation and invasion. HuR as a specific RBP can stabilize p21, p53, and TNF-α for mRNA degradation (Chai et al. 2016).

- **NEAT1/miR-34a-5p/BCL2**

It has been observed that NEAT1 overexpression induced cell proliferation and reduced apoptosis in ovarian cancer cells by suppressing caspase-3 activity. NEAT1 can negatively regulate miR-34a-5p and trigger B-cell lymphoma-2 (BCL2) expression. High expression of NEAT1 could reduce the G0/G1 phase and accelerate the proportion of cells in the S phase. Therefore, knockdown of NEAT1 decreased cell proliferation and increased apoptosis (Ding et al. 2017).

- **NEAT1/miR-382-3p/ROCK1**

A recent study illustrated that high expression of NEAT1 in ovarian patients was correlated to poor prognosis and shorter survival rate. NEAT1 by sponging miR-382-3p and targeting the expression of Rho associated coiled-coil containing protein kinase 1 (ROCK1) as a metastasis-related gene could enhance ovarian cell migration and invasion. Therefore, miR-382-3p reduced tumor metastasis by targeting the 3′-UTR of ROCK1 (Gan et al. 2018).

- **LIN28B/NEAT1/miR-506**

The aberrant expression of NEAT1 was found in high-grade serous ovarian cancer (HGSOC). LIN28B as an oncogene can bind and stabilize NEAT1 expression. Thus, NEAT1 by suppressing miR-506 could regulate tumor cell growth, proliferation, progression, and cell–cell adhesion. Therefore, NEAT1 combined with LIN28B can be potent biomarkers for HGSOC (Yong et al. 2018).

- **NEAT1/miR-194/ZEB1**

NEAT1 was reported to decrease miR-194 in paclitaxel (PTX)-resistant ovarian cancer tissues and cell lines. NEAT1 was discovered to sponge miR-194, stimulate ZEB1 expression, and induce EMT and drug-resistant phenotype. NEAT1 knockdown accelerated cell sensitivity to PTX via promoting PTX-induced apoptosis in vitro. Furthermore, NEAT1 knockdown decreased resistance to drug treatment in vivo (An et al. 2017).

- **NEAT1/miR‑4500/BZW1**

It has been invested that high expression of NEAT1 can inhibit miR‑4500 and stimulate basic leucine zipper and W2 domain‑containing protein 1 (BZW1) in ovarian cancer cells. NEAT1 knockdown was reported to suppress ovarian cancer cell proliferation, colony formation, migration, and glycolysis, and trigger apoptosis (Xu et al. 2020b).

- **NEAT1/miR-365/FGF9**

It has been demonstrated that NEAT1 and FGF9 are overexpressed in ovarian cancer cells. NEAT1 was found to target VEGF, Ang-1, and MMP2 to induce angiogenesis. Overexpression of miR-365 or knockdown of NEAT1 or FGF9 decreased cell proliferation, colony formation, and angiogenesis. The effect of FGF9 knockdown can be reversed by overexpression of NEAT1 or knockdown of miR-365 (Yuan et al. 2021).

- **NEAT1/let-7/ATGL/MEST**

In ovarian cancer cells, NEAT1 expression was upregulated, whereas let-7 g was decreased. NEAT1 is competitively binding to let-7 g and thereby downregulating its expression. Let-7 g suppressed the expression of mesoderm specific transcript (MEST), increased adipose triglyceride lipase (ATGL) production, and decreased cancer cell proliferation, migration, and invasion. Furthermore, silencing of NEAT1 reduced the xenograft tumor growth (Yin and Wang 2021).

### Endometrial cancer

- **NEAT1/IGF1**

In endometrial endometrioid adenocarcinoma (EEC), high expression of NEAT1 was correlated with FIGO stage and lymph node metastasis. Besides, NEAT1 increased cell growth, colony formation, migration, and invasion by increasing the S-phase cells and suppressing apoptosis. This lncRNA was found to enhance the expression of MMP-2, MMP-7, c-myc, and insulin like growth factor 1 (IGF1). NEAT1 also reduced the expression of tissue inhibitor of metalloproteinases 2 (TIMP2) and Cadherin 1. Therefore, siNEAT1 could induced G0/G1 arrest and stimulate cell apoptosis and tumor development (Li et al. 2016).

- **NEAT1/miR-124-3p**

It has been reported that the expression of NEAT1 in endometrial cancer is elevated. NEAT1 by targeting miR-124-3p presented an essential role in tumor development. Therefore, silencing of NEAT1 reduced cell proliferation, migration, and invasion, and promoted apoptosis (Yuan et al. 2022).

- **NEAT1/miR-146b-5p/Wnt3a/HMGA1**

In endometrial cancer cells, the expression level of NEAT1 increased and positively interacted with lymphoid enhancing factor 1 (LEF1), c-myc, and MMP9 through the Wnt/β-catenin signaling. NEAT1 was found to suppress miR-146b-5p, increase the number of cells in the S stage, and decrease the activation of G0/G1 phase-related cycle regulators. Therefore, NEAT1 by targeting the Wnt/β-catenin signaling enhanced cell growth and colony formation in endometrial cancer cells (Huang et al. 2019).

- **NEAT1/miR-144-3p/EZH2**

High expression of NEAT1 was found in endometrial cancer tissues and cell lines. NEAT1 by sponging miR-144-3p can interact with EZH2 as a target gene of miR-144-3p to induce cell growth, colony formation, and invasion. NEAT1 knockdown has been shown to reduce cell proliferating, migrating, and invading. Besides, overexpression of miR-144-3p inhibited tumor cell growth and invasion (Wang et al. 2019c).

- **NEAT1/miR-361/STAT3**

NEAT1 expression was significantly increased in early-stage endometrial cancer tissue samples, and high NEAT1 expression was associated with poor prognosis. NEAT1 was invested to inhibit miR-361 and mediate the expression of STAT3 as an oncogene to stimulate cell proliferation, invasion, and sphere formation. Besides, NEAT1 was reported to stimulate several tumor microenvironment-related genes such as MEF2D, ROCK1, WNT7A, VEGFA, PDE4B and KPNA4. In aggressive endometrial cancer cells, NEAT1 suppression using shRNAs reduced cell proliferation, invasion, sphere formation, and xenograft tumor growth while improving TX responsiveness (Dong et al. 2019).

- **NEAT1/miR-214-3p/Wnt/β-catenin**

In endometrial cancer tissues, NEAT1, HMGA1, and β-catenin were all considerably elevated, while miR-214-3p was dramatically downregulated. NEAT1 increased the mRNA levels of Wnt/β-catenin to stimulate the expression of c-myc and MMP9, thereby accelerating cell proliferation, migration, and invasion in HEC-1A cells. Overexpression of miR-214-3p decreased HEC-1A cell proliferation, motility, and invasion, whereas overexpression of NEAT1 restored these effects. Overexpression of miR-214-3p decreased the activity of the Wnt/β-catenin pathway, whereas overexpression of NEAT1 reversed this effect (Wang et al. 2017).

- **NEAT1/miR-202-3p/TIMD4**

In endometrial tumor and cell lines, overexpression of NEAT1 was related to cell proliferation, migration, and invasion by suppressing miR-202-3p. Depletion of NEAT1 was reported to improve apoptosis and reduce cell progression. NEAT1 could facilitate T cell immunoglobulin and mucin domain 4 (TIMD4) expression as a downstream target of miR-202-3p. Therefore, NEAT1 may provide a potential method for patients with endometrial tumor (Xu et al. 2020c).

- **NEAT1/miR-26a/b-5p/STAT3/YKL-40**

A recent study revealed that cancer-associated fibroblast cells (CAFs)-derived exosomal NEAT1 can target the growth, metastasis, and progression of endometrial cancer. NEAT1 was found to inhibit miR-26a/b-5p and accelerate STAT3 and Chitinase 3-like 1 (YKL-40/CHI3L1) expression levels in endometrial tissues. YKL-40 acts as an oncogene that can be positively regulated by STAT3. Therefore, NEAT1 promoted tumor growth and tumorigenicity in vitro and in vivo (Fan et al. 2021).

## Clinical potential of the lncRNA NEAT1 in gynecologic cancers

In order to enhance therapeutic rates and decrease mortality, early detection, diagnosis along with early therapy are suggested for patients suffering from cancer. Since the evolution of imaging techniques, numerous screening tools have been used to figure out and recognize gynecologic cancers, including mammography, magnetic resonance imaging (MRI), positron emission tomography (PET), computed tomography (CT), and single-photon emission computed tomography (SPECT) (Jafari et al. 2018). Nonetheless, the susceptibility and specificity of such imaging methods have faced some challenges for clinical application. Interestingly, a considerable number of lncRNAs are shown with promising potential for helping diagnosis, prediction of prognosis, and treatment of gynecologic cancers (Hashemipour et al. 2021). Among them, the clinical significance of NEAT1 is evaluated in several studies and the findings have suggested this lncRNA with applications in diagnosis and treatment of patients with gynecological cancers.

Aberrant expression of NEAT1 in serum samples of breast cancer patients is correlated with clinicopathological characteristics, including pathological types, tumor size, histological grading, TNM stage, and hormonal status. Therefore, NEAT1 serum upregulation can predict lower survival rates and poor prognosis in those patients (Zhao et al. 2017; Liu et al. 2020; Swellam et al. 2021; El-Fattah et al. 2021; Zhang et al. 2017; Li et al. 2017). Moreover, NEAT1 sensitivity was shown to be more pronounce for identification of those patients with high-risk factors. Notably, serum expression level of NEAT1 has shown acceptable diagnostic power in differentiation of breast cancer patients from healthy individuals with an area under curve (AUC) value of 0.83 (CI = 0.73 to 0.93, *p* < 0.0001) (El-Fattah et al. 2021). Therefore, lncRNA detection in body fluid is recommended for high-risk patients who were shown to be correlated with hormonal receptors (Swellam et al. 2021; Wang et al. 2021).

Recently, chemoresistance-correlated lncRNAs might be possible markers to speculate cancer cell chemotherapeutic response (Ye et al. 2022). NEAT1 knockdown is implicated in cancer cell sensitization to chemotherapy, and attenuation of SOX2^+^ cancer cell population (Shin et al. 2019a). SOX2 stands as a stemness gene, which modulated cancer initiating and drug-resistant behavior (Song et al. 2016). This is also shown for resistance to 5-fluorouracil (5-FU), cisplatin and Taxol, where NEAT1 downregulation using specific small interfering RNA (siRNA) improved chemosensitivity (Li et al. 2017; Shin et al. 2019b). Thereby, the role of NEAT1 in chemoresistance and cancer stemness proposed that NEAT1 could be applied as a promising clinical curative target for patients with drug resistance (Shin et al. 2019a). Moreover, NEAT1 in combination with specific pathways, such as miR-129/ZEB2 and miR-211/HMGA2 are participated in the chemotherapy drug resistance in cancer cells (Jin et al. 2021). Novel diagnostic technologies, such as microarrays, RNA sequencing (RNA-seq), and qRT-PCR have been used to quantify lncRNAs (Bolha et al. 2017). Currently, investigating the novel drugs targeting lncRNAs has made some advance. Some small molecule inhibitors, siRNAs, antisense oligonucleotides, and CRISPR-Cas9 have been progressed, and indirect regulators of lncRNAs are also pointed out a new window in drug advancement (Malih et al. 2016). Accumulating researches indicate that the CRISPR-Cas9 genome editing technology can be applied to silence lncRNAs (Jin et al. 2021; Zhu et al. 2016). This technology can omit genomes at accurate locations with a special size and high fidelity (Najafi et al. 2022). NEAT1 expression translationally modulated NAD(P)H: quinone oxidoreductase 1 expression in radiation-resistant cancer cells. Suppression of NEAT1 expression by CRISPR-Cas9 has been shown to induce the radiation-resistant cancer cell sensitivity to radiation and decrease cancer cell proliferation and the expression of stem cell markers (Lin et al. 2020). These findings suggest that CRISPR-Cas9 genome editing can be applied for targeting of deregulated NEAT1 in gynecologic cancers (Jin et al. 2021). Taken together, along with an increasing number of lncRNAs, NEAT1 is also suggested with potentials in prediction of prognosis, helping diagnosis and a therapeutic target for patients with gynecologic cancers.

## Conclusion

Considering the above-mentioned examples, we highlighted the recently reported function of NEAT1 in gynecologic cancers. Although the expression of NEAT1 was found to be increased in cancer cells, the exact roles of NEAT1 remain largely unknown and further studies are required to confirm this hypothesis. Based on the literature, it has been suggested that some miRNAs such as miR-124, miR-146b-5p, and miR-34a may be potent targets for gynecologic cancer diagnosis and pre-treatment. Besides, targeting some transcription factors, including STAT3, Wnt, β-catenin, and ZEB1 might be a suitable strategy against gynecologic cancers. Taken together, NEAT1 can be used as a potential biomarker for the treatment of gynecologic cancers.

## Data Availability

The datasets used and/or analyzed during the current study are available from the corresponding author on reasonable request.

## Abbreviations

ATGL: Adipose triglyceride lipase
BZW1: Basic leucine zipper and W2 domain‑containing protein 1
BCL2: B-cell lymphoma-2
BRCA1: Breast cancer susceptibility gene 1
CAFs: Cancer-associated fibroblast cells
CHI3L1: Chitinase 3-like 1
CT: Computed tomography
CPT: Camptothecin
CCND1: Cyclin D1
CPT1A: Carnitine palmitoyltransferase 1A
ceRNA: Competing endogenous RNA
EVs: Extracellular vesicles
EZH2: Enhancer of zeste 2 polycomb repressive complex 2 subunit
EEC: Endometrial endometrioid adenocarcinoma
FUS: Sarcoma
FOXA1: Forkhead box A1
HGSOC: High-grade serous ovarian cancer
HMGA2: High mobility group A2
IGF1: Insulin like growth factor 1
KLF12: Krüppel-like factor 12
LEF1: Lymphoid enhancing factor 1
LncRNAs: Long ncRNA
MEST: Mesoderm specific transcript
MMP2: Metallopeptidase 2
MEN: Multiple endocrine neoplasia
NF-κB: Nuclear factor kappa B
ncRNAs: Non-coding RNAs
NEAT1: Nuclear enriched autosomal transcript 1
PTEN: Phosphatase and tensin homolog
POU2F1: POU class 2 homeobox 1
PET: Magnetic resonance imaging
PTX: Paclitaxel
PET: Positron emission tomography
RRM2: Ribonucleotide reductase regulatory subunit M2
ROCK1: Rho associated coiled-coil containing protein kinase 1
RBP: RNA binding protein
SPECT: Single-photon emission computed tomography
SOX4: SRY-Box transcription factor 4
si-NEAT1: Small interfering RNA against NEAT1
sncRNA: Small non-coding RNA
STAT3: Signal transducer and activator of transcription 3
TJP3: Tight junction protein three
TPD52: Tumor protein D52
TIMP2: Tissue inhibitor of metalloproteinases 2
TIMD4: T cell immunoglobulin and mucin domain 4
TIMM17A: Translocase of inner mitochondrial membrane 17 homolog A
ZO-1: Zonula occludens-1
ZFX: Zinc finger protein X-linked
ZEB1: Zinc finger E-box binding homeobox 1
